# Dietary supplementation with citrus peel extract in transition period improves rumen microbial composition and ameliorates energy metabolism and lactation performance of dairy cows

**DOI:** 10.1186/s40104-024-01110-8

**Published:** 2024-11-09

**Authors:** Lingxue Ju, Qi Shao, Zhiyuan Fang, Erminio Trevisi, Meng Chen, Yuxiang Song, Wenwen Gao, Lin Lei, Xinwei Li, Guowen Liu, Xiliang Du

**Affiliations:** 1 State Key Laboratory for Diagnosis and Treatment of Severe Zoonotic Infectious Diseases, Key Laboratory for Zoonosis Research of the Ministry of Education, Institute of Zoonosis, College of Veterinary Medicine, Jilin University, Changchun, 130062 China; 2 Department of Animal Sciences, Food and Nutrition, Faculty of Agriculture, Food and Environmental Science, Università Cattolica del Sacro Cuore, Piacenza, 29122 Italy

**Keywords:** Adipose tissue, Citrus peel extract, Energy metabolism, Liver, Rumen microbiota

## Abstract

**Background:**

During the transition period, excessive negative energy balance (NEB) lead to metabolic disorders and reduced milk yield. Rumen microbes are responsible for resolving plant material and producing volatile fatty acids (VFA), which are the primary energy source for cows. In this study, we aimed to investigate the effect of citrus peel extract (CPE) supplementation on rumen microbiota composition, energy metabolism and milk performance of peripartum dairy cows.

**Methods:**

Dairy cows were fed either a basal diet (CON group) or the same basal diet supplemented with CPE via intragastric administration (4 g/d, CPE group) for 6 weeks (3 weeks before and 3 weeks after calving; *n* = 15 per group). Samples of serum, milk, rumen fluid, adipose tissue, and liver were collected to assess the effects of CPE on rumen microbiota composition, rumen fermentation parameters, milk performance, and energy metabolic status of dairy cows.

**Results:**

CPE supplementation led to an increase in milk yield, milk protein and lactose contents, and serum glucose levels, while reduced serum concentrations of non-esterified fatty acid, β-hydroxybutyric acid, insulin, aspartate aminotransferase, alanine aminotransferase, and haptoglobin during the first month of lactation. CPE supplementation also increased the content of ruminal VFA. Compared to the CON group, the abundance of Prevotellaceae, Methanobacteriaceae, Bacteroidales_RF16_group, and Selenomonadaceae was found increased, while the abundance of Oscillospiraceae, F082, Ruminococcaceae, Christensenellaceae, Muribaculaceae UCG-011, Saccharimonadaceae, Hungateiclostridiaceae, and Spirochaetaceae in the CPE group was found decreased. In adipose tissue, CPE supplementation decreased lipolysis, and inflammatory response, while increased insulin sensitivity. In the liver, CPE supplementation decreased lipid accumulation, increased insulin sensitivity, and upregulated expression of genes involved in gluconeogenesis.

**Conclusions:**

Our findings suggest that CPE supplementation during the peripartum period altered rumen microbiota composition and increased ruminal VFA contents, which further improved NEB and lactation performance, alleviated lipolysis and inflammatory response in adipose tissue, reduced lipid accumulation and promoted gluconeogenesis in liver. Thus, CPE might contribute to improve energy metabolism and consequently lactation performance of dairy cows during the transition period.

**Supplementary Information:**

The online version contains supplementary material available at 10.1186/s40104-024-01110-8.

## Background

The peripartum period in dairy cows spans from gestation to lactation, and is considered a critical phase that affects milk yield and energy metabolism [1]. During this phase, the dry matter intake and thus nutrient supply of dairy cows can decline or fail to meet the rapidly increasing energy requirements [2, 3], which causes negative energy balance (NEB) [4]. To cover the energy shortage, cows mobilize lipids from adipose tissue, resulting in a significant release of non-esterified fatty acid (NEFA) [5]. An excessive influx of NEFA into the liver can lead to inadequate oxidation, promoting the marked synthesis of β-hydroxybutyric acid (BHBA) and the accumulation of triglycerides (TG) into the liver, which can potentially elicit lipotoxicity and causes hepatic injury and steatosis [6]. Moreover, inflammatory phenomena exacerbate lipid mobilization of adipose tissue and induce excessive release of NEFA, which further leads to liver injury [7, 8].

In dairy cows, fatty liver and ketosis occur as a consequence of NEB during intensive lactation after calving. The nutrients produced by rumen microbes can meet 70% of energy requirements of the ruminants [9], which makes them essential for host energy metabolism [10]. Dairy cows mainly use propionic acid produced by rumen microbial fermentation to synthesize glucose via gluconeogenesis in hepatocytes [11]. It is known that the rumen microbiota directly affects the volatile fatty acid (VFA) levels [12]. Moreover, previous studies have indicated that an increased absorption of VFA can alleviate liver damage [13] and reduce adipocyte lipolysis [14]. Previous studies have shown that composition and abundance of rumen microbiota in dairy cows fluctuate during the dry period and lactation [15–17]. It has been reported that rumen VFA contents and several microbial genera, such as *Prevotella*, *Ruminococcus*, *Selenomonas*, and *Succinivibrio*, were diminished in the rumen of dairy cows with hyperketonemia [18, 19]. Thus, modulating the rumen microbiota may contribute to improve metabolic diseases in dairy cows, like fatty liver and ketosis.

Natural extracts are widely used as feed additives due to their low toxicity and vast source availability [20–22]. For instance, grape seed extract and inulin can improve lactation performance of dairy cows [23, 24]. Citrus peel extract (CPE) is one of the common natural extracts, which is rich in flavonoids, such as hesperidin [25]. It has been reported that hesperidin has anti-inflammatory effect in various animal models and cell types, such as rat synoviocyte and murine macrophage [26, 27]. Therefore, CPE is considered to be used as additive in forage [28].

In mice fed a high-fat diet, administration of hesperidin altered the intestinal microbiota composition, and thus mitigating liver damage and lipid accumulation [29]. In dairy cows, CPE supplementation has been shown to increase milk yield and the proportion of unsaturated fatty acids of conjugated linoleic acid [30]. Additionally, CPE inhibited ammoniagenesis and improved nitrogen utilization in an in vitro rumen batch refermentation system [31]. Notably, the citrus peel flavonoids extract has been found to alter hindgut microbiota composition and reduce serum inflammatory cytokines in dairy cows fed a high-starch diet [32]. However, the effects of CPE on rumen microbiota, energy metabolism and lactation performance of dairy cows during the transition period remain underexplored. In the present study, we hypothesized that dietary supplementation with CPE could contribute to alleviate NEB and improve lactation performance by modulating rumen microbiota composition. Hence, we explored the effects of CPE on rumen microbiota, glucose, and lipid metabolism in adipose tissue and liver, as well as milk yield in dairy cows during the transition period.

## Materials and methods

### Animals and experimental design

**Fig. 1:**
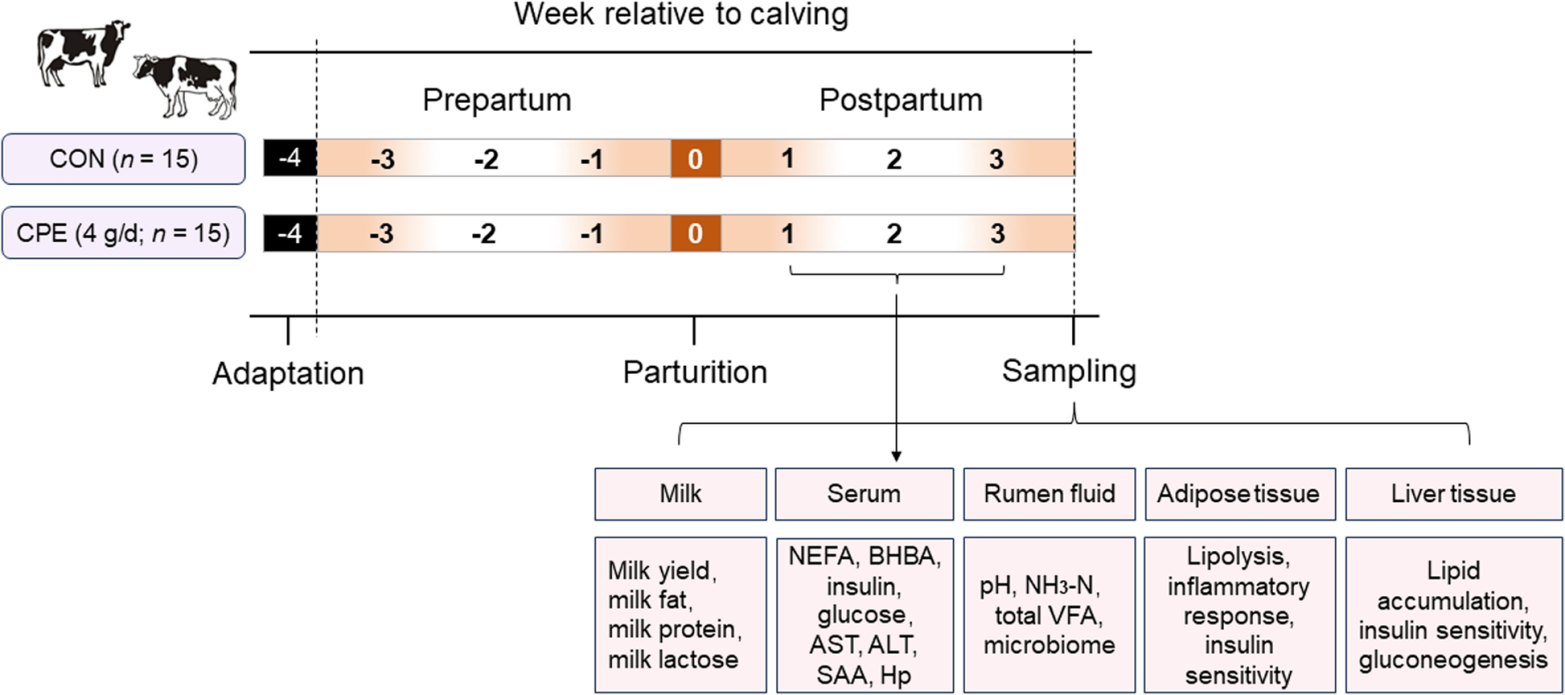
The experimental design. Cows were randomly allocated to two groups, which included a CON group and a CPE treatment group. Dissolved CPE (4 g/d) was given by intragastric administration before morning feeding. Experiment began 21 d before expected calving and ended 21 d after calving. On the last day of the experiment, rumen fluid, milk, serum, liver and subcutaneous adipose tissues were collected

Experimental procedures in this experiment were approved by the Ethics Committee on the Use and Care of Animals of Jilin University (Changchun, China, SY202303302). The experimental period lasted 6 weeks, including 21 d before and 21 d after parturition. Dairy cows of similar parity (median = 3, range = 2–4) and bodyweight (657.8 ± 48.1 kg, mean ± standard deviation) selected from the same dairy farm were enrolled in this study. Cows were fed 3 times a day at 0700, 1300 and 1900. All cows had free access to water and total mixed ration diet. After 1 week of acclimation, cows were randomly allocated to two groups, which included a control (CON) group and a CPE treatment group. Dissolved CPE (4 g/d) was given by intragastric administration before morning feeding. The CPE was extracted from citrus peel and dissolved in water (Changsha Tianwei Biotechnology Co., Ltd., Changsha, China; Catalog number: TWE-258). The main component of CPE is hesperidin (> 50% purity), other components including neohesperidin, synephrine, and diosmin. After calving, dairy cows underwent a thorough clinical examination to rule out cows with displaced abomasum, mastitis, endometritis or laminitis. Fifteen cows in each group were recruited for this study. The experimental design is shown in Fig. 1.

### Milk sample collection and analysis

Milk samples were collected at 21 d postpartum. Cows were milked 3 times at 0630, 1230 and 1830 h, and the milk yield was recorded for 3 consecutive days. Samples were pooled proportionately based on milk production and were combined with 0.6 mg/mL potassium dichromate as a preservative, collected into sterile centrifuge tubes and stored at 4 °C for the determination of milk composition. The milk samples were determined for milk fat, milk protein, and milk lactose using Milkoscan FT1 (Foss Electric, Hillerød, Denmark).

### Blood sample collection and analyses

Blood samples were collected at 0700 h (before morning feeding) through caudal vein at d 7, 14, and 21 after calving. The blood samples were placed at room temperature for 2 h and centrifuged at 3,000 × *g* for 15 min. Serum was obtained and stored at −80 °C until analysis. The concentrations of BHBA, NEFA, glucose, aspartate aminotransferase (AST) and alanine aminotransferase (ALT) in serum were measured via a Hitachi 7170 autoanalyzer with commercially-available kits. The concentrations of serum amyloid A protein (SAA), haptoglobin (Hp), and insulin in serum were measured using the enzyme linked immunosorbent assay (ELISA) kit (SAA: CB10135-Bv; Hp: CB10076-Bv; insulin: CB10140-Bv; COIBO BIO, Shanghai, China).

### Liver and adipose tissues sampling

Liver and adipose tissues were collected at 21 d postpartum via biopsy. The surgery places were shaved, sanitized with iodine scrub and 75% alcohol, and anesthetized with a subcutaneous injection of 2% lidocaine HCl. Liver tissues were obtained using the biopsy puncture needle (Shanghai Surgical Instrument Factory, Shanghai, China), inserted into the liver through the intercostal muscle layers. Subcutaneous adipose tissues were collected by blunt dissection through incision in the skin and subcutaneous tissue, and then washed with saline. Samples were immediately quenched in liquid nitrogen and stored at −80 °C or fixed with 4% paraformaldehyde or OCT compound (Sakura Finetek, Torrance, CA, USA) for subsequent analysis.

### Histopathology

Liver tissue samples from each cow were fixed in 4% paraformaldehyde neutral buffer solution, embedded in paraffin, sliced into 8 μm sections, and then picked up onto glass slides. These slides were dewaxed with xylene, rehydrated through descending concentrations of alcohol, and stained with hematoxylin–eosin (H&E). As for Oil-Red O staining, liver tissues were frozen in optimal cutting temperature compound (Sakura Finetek), sectioned into 8 μm at −18 °C, stained with Oil-Red O (Sigma-Aldrich, St. Louis, USA), and counterstained with hematoxylin. The histological features were observed and captured at 100× under a light microscope (Olympus, Tokyo, Japan).

### TG content determination

Liver tissue (20 mg) was grinded with an electric high-speed homogenizer in 1 mL of lysis buffer, part of the supernatant was used to determine the total protein concentrations using the Bicinchoninic acid (BCA) assay (P1511, Applygen Technologies Inc., Beijing, China). The remaining supernatant was heated in 70 °C water bath for 10 min, and then was centrifuged at 800 × *g* for 5 min at room temperature. Ten-microliter aliquots of supernatant were taken to mixed with 190 µL of chromogenic liquid, used for TG assay using an enzymatic kit (E1013, Applygen Technologies Inc.) according to the manufacturer’s instructions.

### RNA isolation and quantitative real‑time PCR assay

The total RNA was extracted from liver and adipose tissues using RNAiso Plus (9109, TaKaRa, Dalian, China) according to the manufacturer’s instructions. The ratio of UV activity at 260/280 nm was used to measure the purity of RNA using K5500 Micro-Spectrophotometer (Beijing Kaiao Technology Development Co. Ltd., Beijing, China). The RNA concentrations are measured at 260 nm, and the cDNA were synthesized from 5 µg of purified total RNA using a reverse-transcription kit containing random hexamers and oligo deoxythymidine primers (RR047A, TaKaRa). The SYBR green plus reagent kit (DRR041A, TaKaRa) was used to detect relative mRNA expression of target genes on a 7500 Real-Time PCR System (Applied Biosystems, Foster City, USA). The relative expression of each target gene was calculated using the 2^−∆∆Ct^ method. All primers of target genes (Table S1) were designed using Primer Express software (Applied Biosystems) and synthesized by Sangon Biotech Co. Ltd., Shanghai, China.

### Protein extraction and Western blot assay

Liver and adipose tissue was cut into pieces before protein extraction. Total proteins were extracted and prepared for Western blotting using a commercial kit according to the manufacturer’s instructions (P0013B, Beyotime Institute of Biotechnology, Jiangsu, China). The BCA assay was performed to determine protein content of the homogenates according to the supplier’s instructions (P1511, Applygen Technologies Inc.). Twenty µg of total protein were separated in 12% SDS-polyacrylamide gels and transferred into polyvinylidene fluoride membranes (0.45 μm). After blocked with 3% BSA in Tris-HCl (50 mmol/L Tris, pH 7.4, 200 mmol/L NaCl) buffer solution containing 0.1% Tween-20 for 4 h at room temperature, the membranes were incubated overnight at 4 °C with specific antibodies against protein kinase B (AKT; 1:5,000; ab32505), peroxisome proliferator-activated receptor-α (PPARα; 1:5,000; ab126285), perilipin 1 (PLIN1; 1:1,000; ab3526), cell death-inducing DNA fragmentation factor-α-like effector c (CIDEC; 1:1,000; ab198204), adipose triglyceride lipase (ATGL; 1:1,000; ab99532), β-actin (1:2,000; ab8226; Abcam, Cambridge, USA), phosphorylated-AKT (p-AKT; 1:2000; #4060), hormone-sensitive lipase (HSL; 1:1,000; #4107), p-HSL (1;1,000; #4139), nuclear factor kappa-B (NF-κB; 1:1,000; #4764), p-NF-κB (1:1,000; #3033), inhibitor of NF-κB (IκBα; 1:1,000; #4814; Cell Signaling Technology Inc., Danvers, USA), and sterol regulatory element-binding protein 1c (SREBP-1c; 1:500; NB100–2215; Novus Biologicals, Littleton, USA) respectively. Subsequently, the membranes were washed 3 times in the Tris-buffered saline and Tween buffer and then incubated with an appropriate secondary antibody at room temperature for 45 min. Immunoreactive bands were visualized via a Tanon Imaging System (Tanon 4600, Shanghai, China) using an enhanced chemiluminescence solution (WBULS0500, Millipore, Bedford, USA). All bands were analyzed using Image-Pro Plus 6.0 (Media Cybernetics, Rockville, USA) and β-actin was used as an internal control.

### Rumen fluid sampling and measurement

Rumen fluid samples were collected 3 h after morning feeding on the last day of experiment by using a rumen fluid sampler (A1141K, Colebo Equipment Co., Ltd., Wuhan, China). The first 2 tubes of rumen fluid were discarded to avoid saliva contamination, and approximately 100 mL rumen fluid sample was collected from each cow. Each sample was filtered through 4 layers of sterile gauze, and the ruminal pH was immediately measured using a portable pH meter (Thermo Fisher Scientific, Waltham, USA). Filtrates were used to analyze the concentrations of total VFA, acetate, propionate, butyrate, ammonia nitrogen (NH_3_-N), and ruminal microorganisms. Rumen NH_3_-N was determined using phenol-hypochlorite assay by visible spectrophotometry [33]. The concentration of total VFA, acetate, propionate, and butyrate were analyzed using a gas chromatography (6890 N; Agilent technologies, Avondale, PA, USA).

#### Ruminal microorganism DNA extraction, PCR amplification and sequencing

Total microbial DNA from 30 rumen fluid samples were extracted according to the instructions of the E.Z.N.A.^®^ Soil DNA Kit (Omega Bio-Tek, Norcross, USA). DNA integrity and concentration was assessed by 1% agarose gel electrophoresis, and stored at −80 °C for further use. The 16S V3–V4 regions were amplified as previously reported [34]. Rumen samples were used as templates with the primer pairs 338F (5′-ACTCCTACGGGAGGCAGCAG-3′) and 806R (5′-GGACTACHVGGGTWTCTAAT-3′) using T100 Thermal Cycler PCR thermocycler (BIO-RAD, Hercules, USA). The thermocycling conditions involved a 3 min denaturation at 95 °C, followed by 27 cycles including 30 s of denaturation at 95 °C, 30 s of annealing at 55 °C, 45 s of extension at 72 °C, and 10 min final extension step at 72 °C. PCR products were extracted from 2% agarose gels and purified using the PCR Clean-Up Kit (YuHua, Shanghai, China). Analysis of microbiota was performed by 16S rRNA sequencing using Illumina Miseq (PE250) sequencing platform (Illumina, San Diego, USA) according to the standard protocols by Majorbio Bio-Pharm Technology Co., Ltd. (Shanghai, China). The raw sequences from 16S rRNA sequencing for this study are available at the National Center for Biotechnology Information (NCBI) Sequence Read Archive (SRA) under the BioProject accession numbers PRJNA1119431 (https://www.ncbi.nlm.nih.gov/).

#### Correlation analysis

The correlation analysis was conducted using the platform of Majorbio (https://cloud.majorbio.com). The correlations between rumen microbiome and rumen fermentation parameters, lactation performance, metabolic parameters and hepatic TG were calculated by Spearman’s correlation coefficient. The range of the correlation coefficient (*r*) was from −1 to 1. *r* > 0 and *r* < 0 represented a positive correlation and negative correlation. The |*r*| value denoted the degree of correlation between variables. Correlation significance *P*-values below 0.05 and 0.01 were regarded as significant and highly significant correlations.

### Statistical analysis

Bioinformatic analysis of the rumen fluid samples were carried out using the Majorbio Cloud platform (https://cloud.majorbio.com). The alpha diversity including Shannon, Simpson, Sobs, ACE and Chao were compared with Mothur (v1.30.1). Beta diversity was determined to compare the similarity and dissimilarities between CON and CPE group by principal component analysis (PCA) and principal coordinate analysis (PCoA) based on Bray-curtis dissimilarity using Vegan v2.5-3 package. ANOVA test using Vegan v2.5-3 package was used to access statistics significance. The linear discriminant analysis (LDA) effect size (LEfSe) was performed to identify the prominently abundant taxa.

Data statistics were performed by SPSS 19.0 software (SPSS Inc., Chicago, USA) or GraphPad Prism 9.0 (GraphPad Software Inc., San Diego, USA). All data were analyzed with unpaired *t*-tests. Results with *P* < 0.05 were considered statistically significant, *P* < 0.01 were considered as a highly significant difference. Data were presented as means ± standard error of the mean (SEM).

## Results

### Effect of CPE supplementation on lactation performance and metabolic parameters

Compared to the CON group, cows supplemented with CPE showed a significant increase in milk yield, as well as in milk protein and lactose contents (Table 1). Additionally, milk fat content was slightly higher in the CPE group compared to the CON group (Table 1). These findings indicate that CPE supplementation improved lactation performance in peripartum dairy cows.

**Table 1:** Effects of CPE supplementation on lactation performance in dairy cows

Item	CON (*n* = 15)	CPE (*n* = 15)	*P*-value
Milk yield, kg/d	42.25	45.71	˂ 0.01
Milk fat, %	3.71	3.85	0.46
Milk protein, %	3.488	3.863	˂ 0.05
Milk lactose, %	5.233	5.576	˂ 0.05

*CON* Control group, *CPE* Citrus peel extract group

**Fig. 2:**
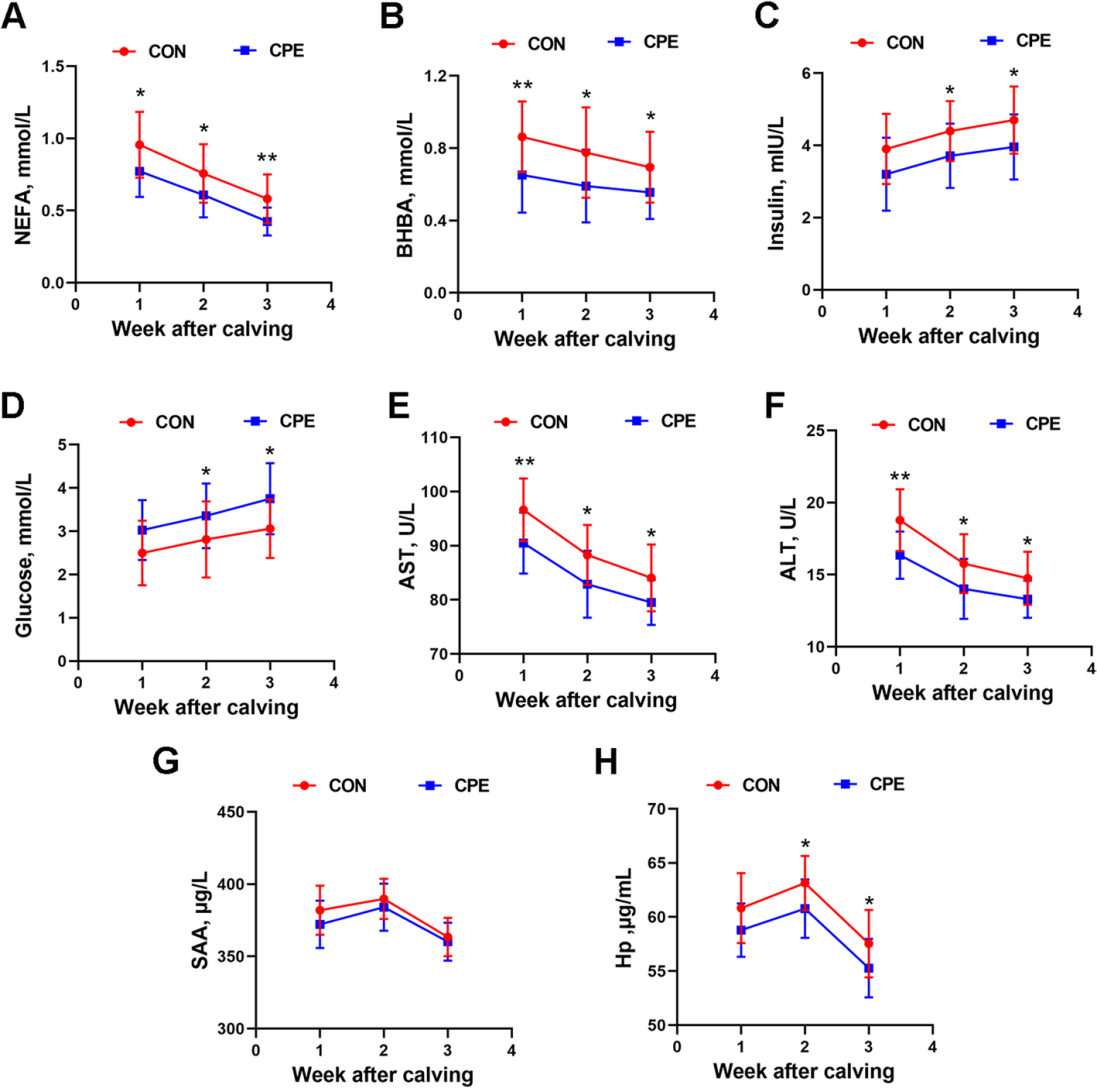
Effect of CPE supplementation on serum metabolic parameters of dairy cows. **A**–**D** The concentrations of NEFA, BHBA, insulin and glucose in the serum. **E** and **F** The activity of AST and ALT in serum. **G** and **H** The content of serum SAA and Hp. Data were analyzed with unpaired *t*-tests and expressed as mean ± SEM. *n* = 15 per group. ^*^P ˂ 0.05, ^**^P ˂ 0.01

Serum levels of NEFA, BHBA, and insulin were significantly lower in the CPE group compared to the CON group (Fig. 2A–C; *P* ˂ 0.05). Conversely, glucose levels were higher in the CPE group (Fig. 2D; *P* ˂ 0.05). Serum indicators of liver function ALT and AST were lower in CPE group (Fig. 2E and F; *P* ˂ 0.05). Although the SAA levels showed no significant differ (Fig. 2G), Hp levels were reduced in the CPE group compared to the CON group (Fig. 2H; *P* ˂ 0.05). These data demonstrate that dietary supplementation with CPE alleviated NEB and reduced the acute phase response in dairy cows.

### Effect of CPE supplementation on rumen fermentation parameters

**Fig. 3:**
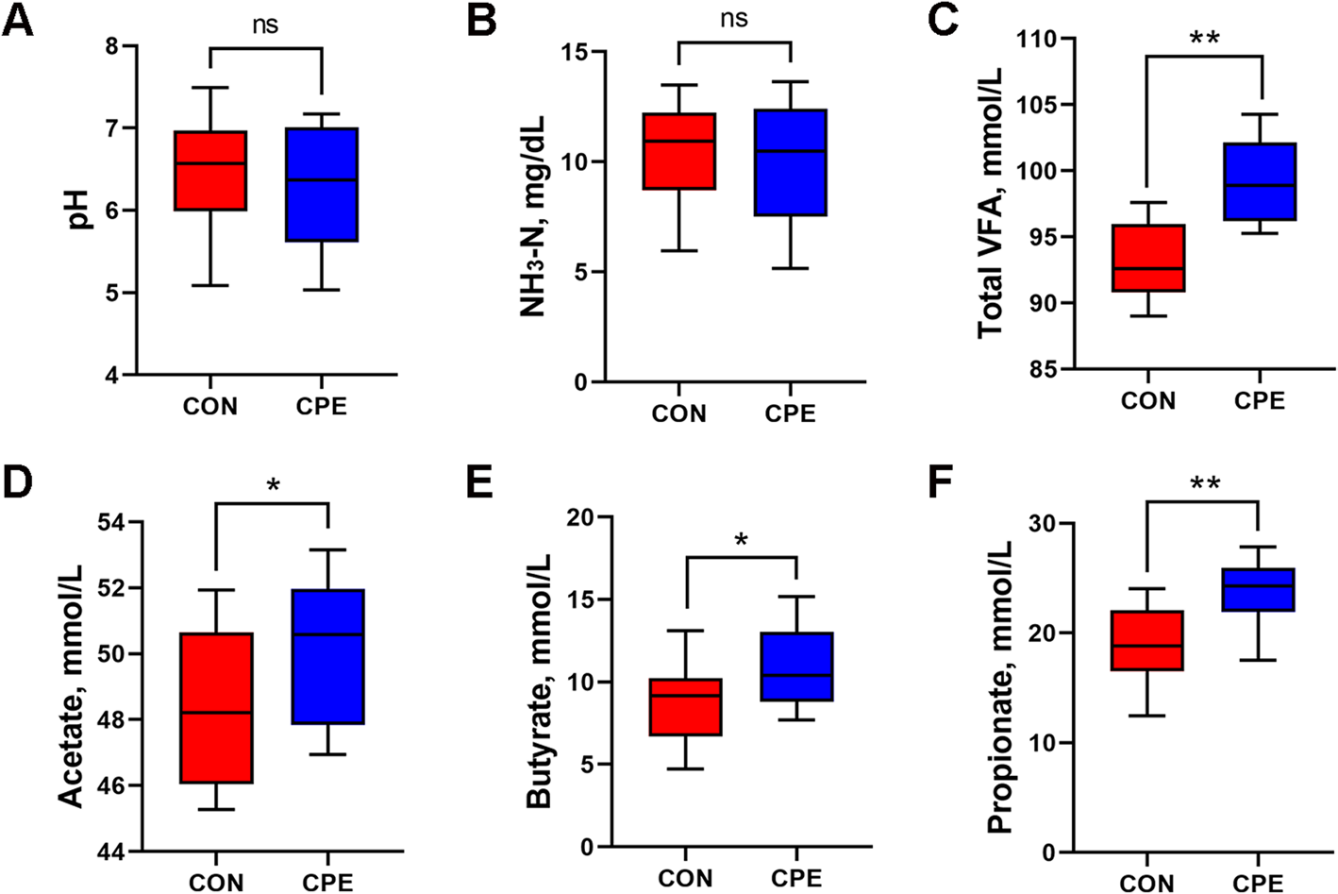
Effect of CPE supplementation on rumen fermentation parameters of dairy cows. **A** Ruminal pH. **B** and **C** The concentrations of NH_3_-N and total VFA in the rumen fluid. **D**–**F** Concentrations of acetate, butyrate, and propionate of rumen fluid among CON and CPE group. *n* = 15 per group. Data were analyzed with unpaired *t*-test and expressed as mean ± SEM. ^*^P ˂ 0.05, ^**^P ˂ 0.01

Three weeks after calving, the ruminal pH and concentration of NH_3_-N did not differ among groups (Fig. 3A and B). However, total VFA contents in the rumen were significantly increased with the addition of CPE (Fig. 3C; *P* ˂ 0.01), along with higher concentrations of acetate, butyrate (Fig. 3D and E; *P* ˂ 0.05), and propionate (Fig. 3F; *P* ˂ 0.01). These observations demonstrate CPE supplementation increased VFA production in the rumen of dairy cows.

### Effect of CPE on rumen microbiota

**Fig. 4:**
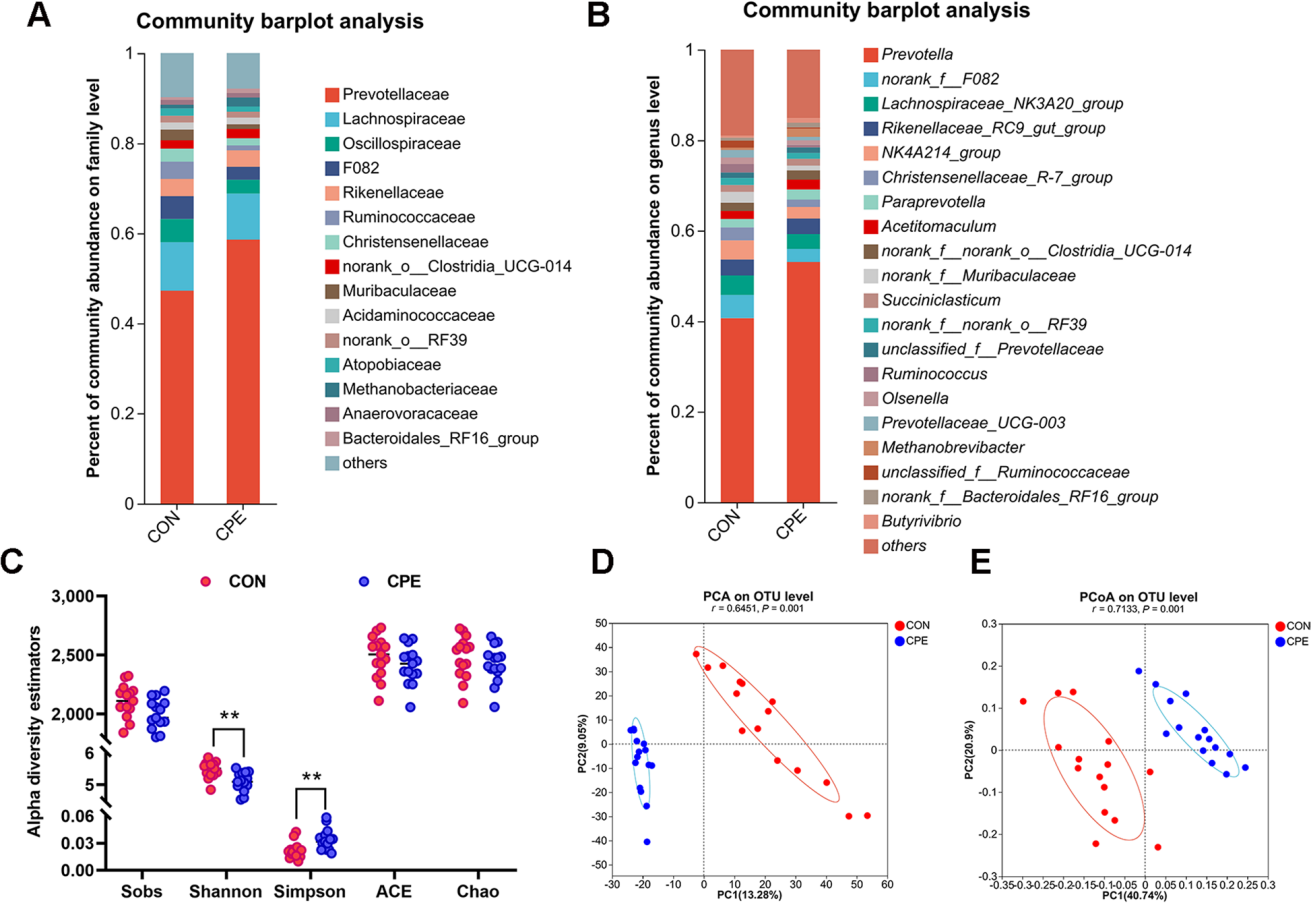
Effect of CPE supplementation on the rumen microbial composition. **A** The composition of rumen microbiome at family level. **B** The composition of rumen microbiome at genus level. **C** Sobs, Shannon, Simpson, ACE, and Chao indexes of ruminal microbiota communities. **D** PCA of ruminal microbiota communities. **E** PCoA of rumen microbial communities. *n* = 15 per group

The impact of CPE supplementation on the rumen microbiota of dairy cows was assessed using 16S rRNA high-throughput sequencing. Although overall bacterial taxa composition was similar between the CON and CPE groups, notable differences were observed for the relative abundance of certain microbes at the family and genus levels (Fig. 4A and B). As shown in Fig. 4C, α-diversity analysis revealed a reduction in the Shannon index (*P* ˂ 0.01) and an increase in the Simpson index (*P* ˂ 0.01) in the CPE group, with trends indicating declines in the Sobs, ACE, and Chao indexes. PCA and PCoA results showed that nodes representing rumen microorganisms were significantly separated in different areas on the coordinate axis (ANOSIM *P* ˂ 0.01), which indicates at the OTU level that CON and CPE groups had a distinct rumen microbial composition (Fig. 4D and E). Taken together, these findings indicate that CPE supplementation reduced richness and diversity of the ruminal microbiota community.

### Differentiation of rumen microbes between the CON and CPE groups

**Fig. 5:**
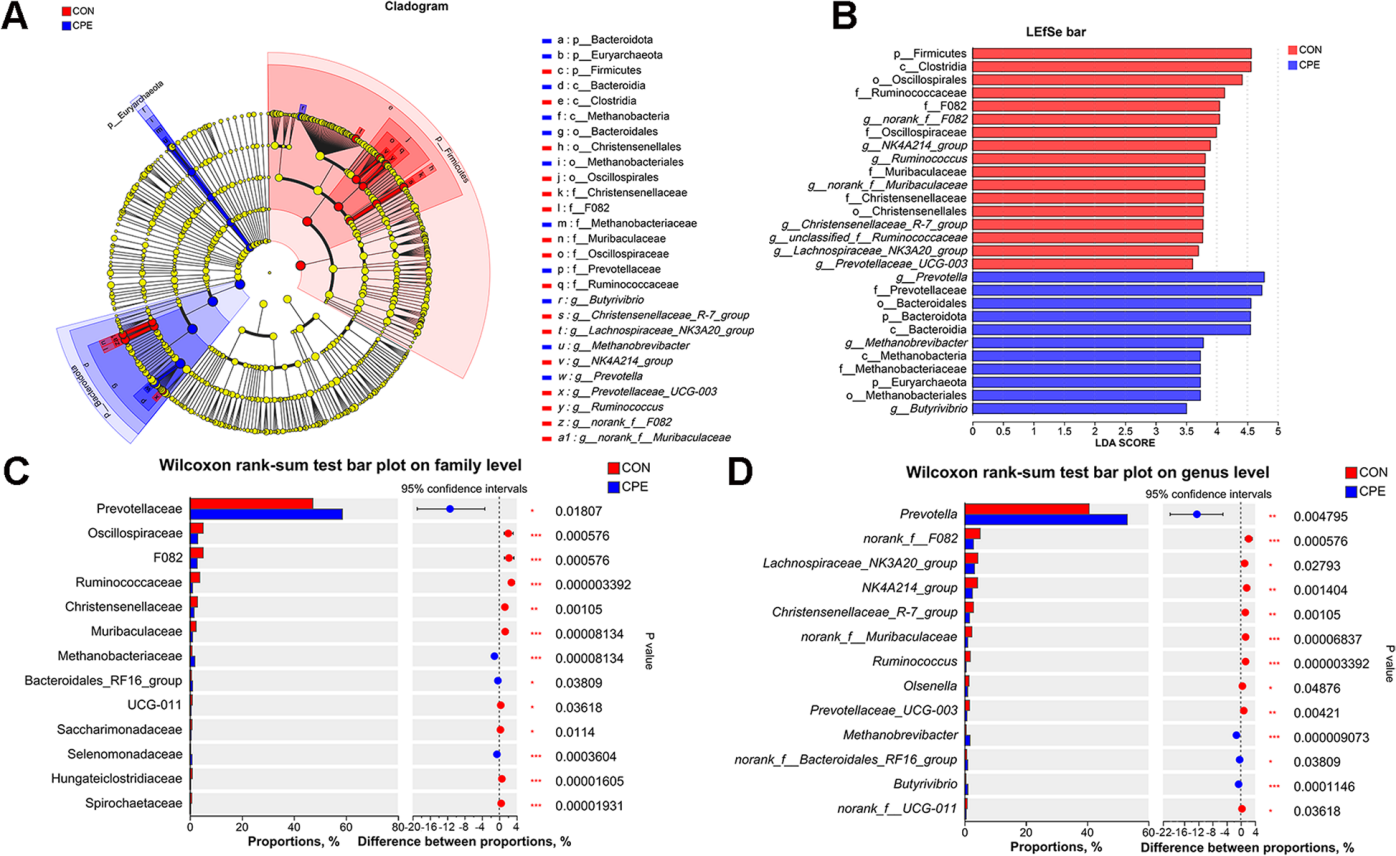
Effect of CPE supplementation on composition difference of rumen microbiota. **A** LEfSe analysis of rumen microbial communities. The nodes in red and blue represent the microbes that are significantly enriched in the corresponding groups and have a significant influence on the difference between CON and CPE group, the yellow nodes represent the microbes that have no significant difference between the 2 groups. **B** LDA discrimination result table of rumen microbial communities. Higher LDA score represents greater importance of the bacteria. **C** The difference of rumen microbiome at family level. **D** The difference of rumen microbiome at genus level. The statistical analysis was performed using the Wilcoxon rank-sum test. *P* ˂ 0.05 and LDA score > 2.5 was considered as a significant difference, *P* ˂ 0.01 was considered as a highly significant difference. *n* = 15 per group

The characteristic ruminal bacteria in each of the two groups were identified using LEfSe and LDA. A total of 28 bacteria taxa were identified in CON and CPE groups (Fig. 5A and B; LDA > 2.5). The cladogram showed significant differences in bacterial populations at the phylum to the genus level (Fig. 5A), and the LDA bar showed the impact of the abundance of each species on the observed differences between CON and CPE groups (Fig. 5B). The Wilcoxon rank-sum test further showed that, at the family level, the abundance of Prevotellaceae, Methanobacteriaceae, Bacteroidales_RF16_group, and Selenomonadaceae (*P* ˂ 0.05) was significantly increased in the CPE group, while the abundance of Oscillospiraceae, F082, Ruminococcaceae, Christensenellaceae, Muribaculaceae, UCG-011, Saccharimonadaceae, Hungateiclostridiaceae, and Spirochaetaceae (*P* ˂ 0.05) was significantly lower compared to the CON group (Fig. 5C). At the genus level, CPE supplementation significantly increased the abundance of *Prevotella*, *Methanobrevibacter*, *norank_f__Bacteroidales_RF16_group* and *Butyrivibrio* (*P* ˂ 0.05), while decreased the abundance of *norank_f__F082*, *Lachnospiraceae_NK3A20_group*, *NK4A214_group*, *Christensenellaceae_R-7_group*, *norank_f__Muribaculaceae*, *Ruminococcus*, *Olsenella*, *Prevotellaceae_UCG-003*, and *norank_f__UCG-011* (Fig. 5D; *P* ˂ 0.05).

### Effect of dietary supplementation with CPE on adipose tissue

**Fig. 6:**
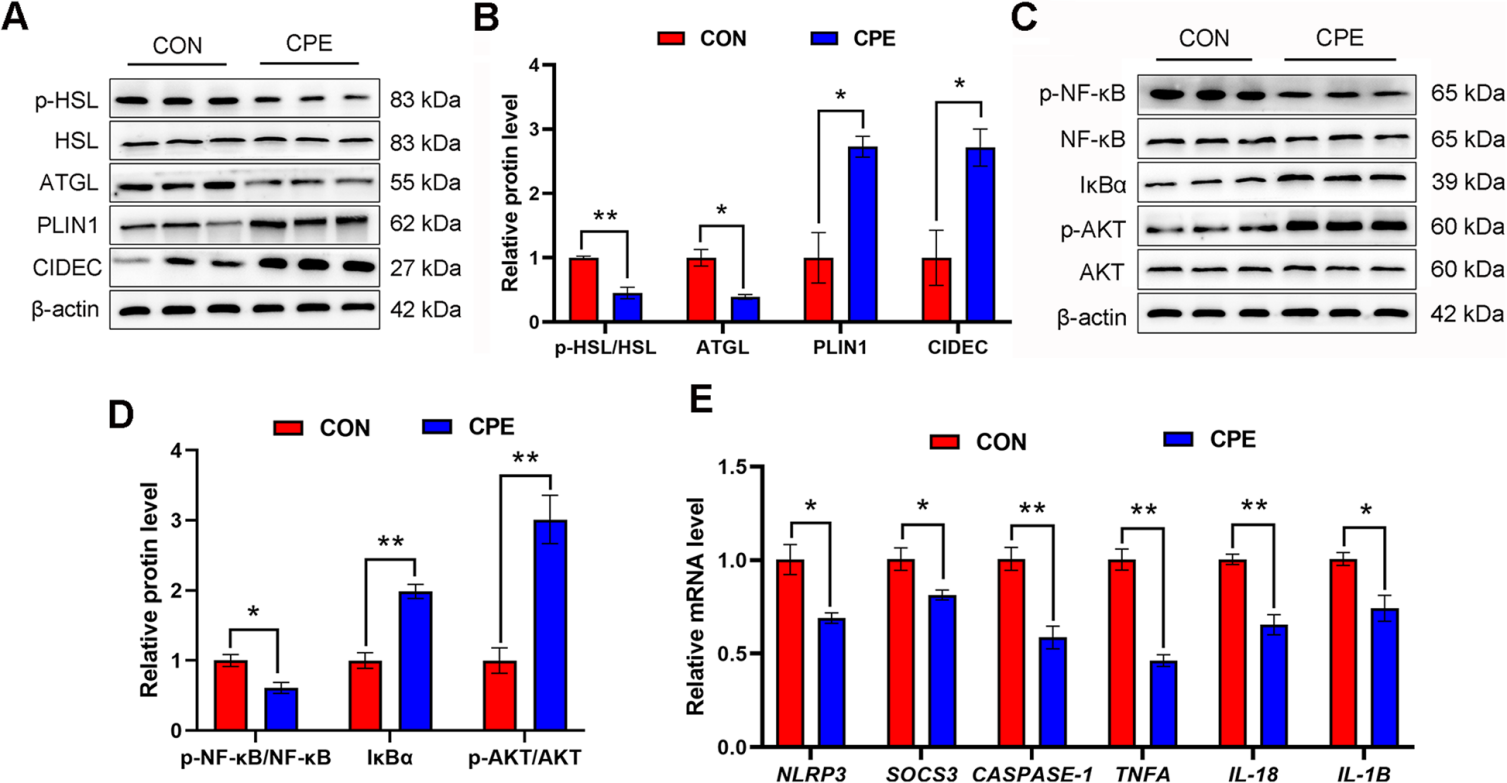
Effect of CPE supplementation on lipolysis, inflammatory response, and insulin sensitivity of adipose tissue. **A** Representative blots of p-HSL, HSL, ATGL, PLIN1, CIDEC and β-actin. **B** Quantification of protein levels of p-HSL, ATGL, PLIN and CIDEC. **C** Representative blots of p-NF-κB, NF-κB, IκBα, p-AKT, AKT and β-actin. **D** Quantification of protein levels of p-NF-κB, IκBα, and p-AKT. **E** Relative mRNA levels of *NLRP3*, *SOCS3*, *CASPASE-1*, *TNFA*, *IL-18* and *IL-1B*. *n* = 15 per group. Data were analyzed with unpaired *t*-tests and expressed as mean ± SEM. ^*^P ˂ 0.05, ^**^P ˂ 0.01

Western blot analysis of adipose tissue lipolysis revealed that the ratio of p-HSL to HSL (*P* ˂ 0.01) and protein abundance of ATGL (*P* ˂ 0.05) were decreased, whereas protein abundance of PLIN1 and CIDEC (*P* ˂ 0.05) were increased in the adipose tissue of cows in the CPE group (Fig. 6A and B). Moreover, protein abundance of p-NF-κB was reduced (*P* ˂ 0.05), while the expression of IκBα (*P* ˂ 0.01) and the ratio of p-AKT to AKT (*P* ˂ 0.01) were increased in the CPE group compared with CON group (Fig. 6C and D). In addition, mRNA abundance of NLR family pyrin domain containing protein 3 (*NLRP3*), suppressor of cytokine signaling 3 (*SOCS3*), cysteinyl aspartate specific proteinase (*CASPASE-1*), tumor necrosis factor-α (*TNFA*), interleukin 18 (*IL-18*), and interleukin 1B (*IL-1B*) was lower in the CPE group (Fig. 6E; *P* < 0.05). Taken together, these results indicate that CPE supplementation alleviated lipolysis and inflammatory response, while enhancing insulin sensitivity in the adipose tissue of dairy cows.

### Effect of dietary supplementation with CPE on liver

**Fig. 7:**
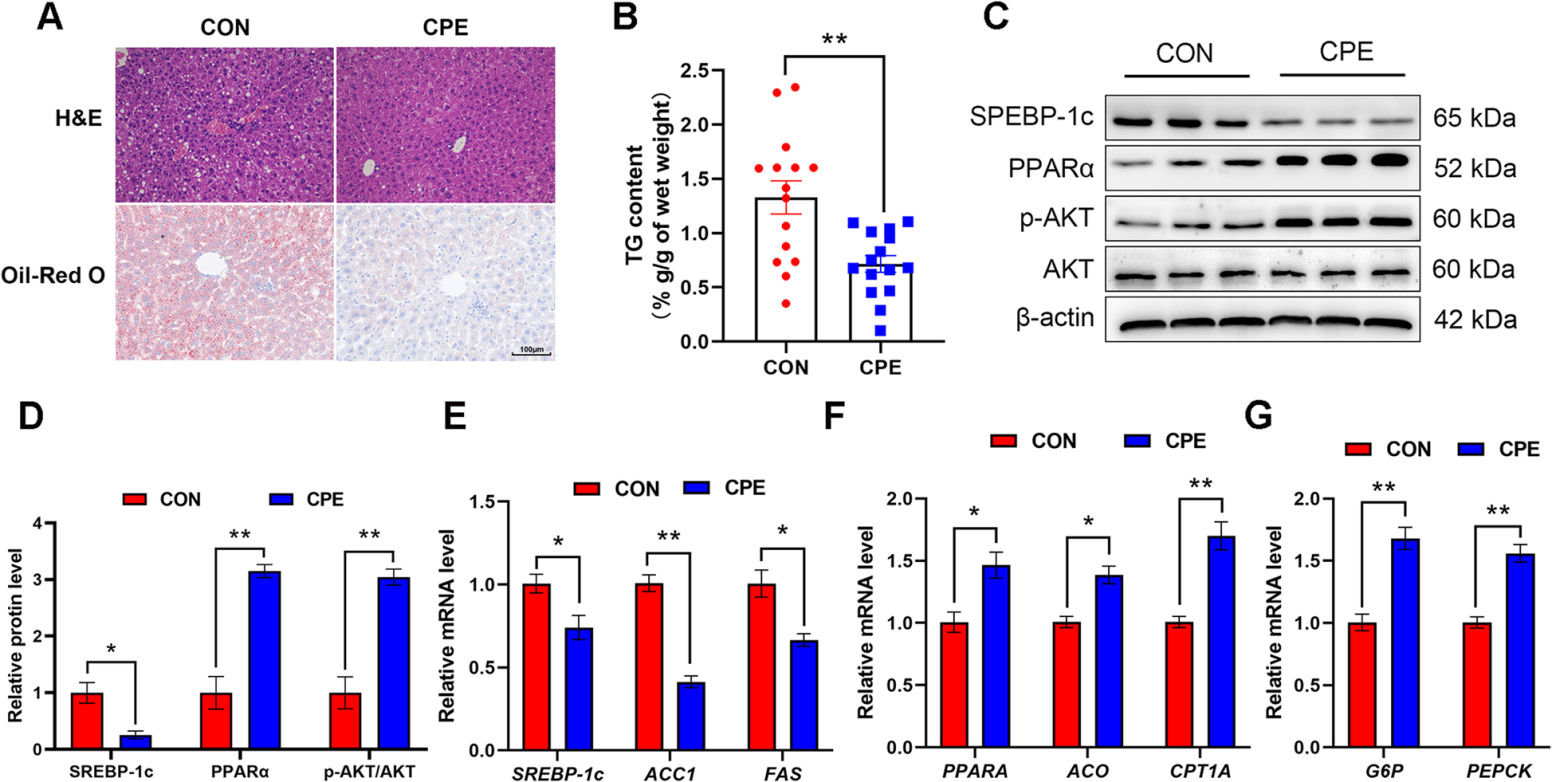
Effect of CPE supplementation on lipid accumulation and insulin sensitivity of liver. **A** H&E and Oil-Red O staining of liver. Scale bar = 100 μm. **B** The content of hepatic TG. **C** Representative blots of SREBP-1c, PPARα, p-AKT, AKT, and β-actin. **D** Quantification of protein levels of SREBP-1c, PPARα, and p-AKT. **E** Relative mRNA levels of *SREBP-1c*, *ACC1*, and *FAS*. **F** Relative mRNA levels of *PPARA*, *ACO*, and *CPT1A*. **G** Relative mRNA levels of *G6P* and *PEPCK*. *n* = 15 per group. Data were analyzed with unpaired *t*-tests and expressed as mean ± SEM. ^*^P ˂ 0.05, ^**^P ˂ 0.01

Histological observations of liver tissue using H&E and Oil-Red O staining, along with the determination of hepatic TG content (*P* < 0.01) showed that CPE supplementation reduced lipid accumulation in the liver of dairy cows (Fig. 7A and B). Compared to the CON group, dietary supplementation with CPE decreased the protein abundance of SREBP-1c (*P* ˂ 0.05), while increasing the protein abundance of PPARα and the ratio of p-AKT to AKT (Fig. 7C and D; *P* ˂ 0.01). Moreover, mRNA abundance of *SREBP-1c*, acetyl CoA carboxylase 1 (*ACC1*) and fatty acid synthase (*FAS*) decreased in the CPE group compared to the CON group, while mRNA abundance of *PPARA*, acyl-CoA oxidase (*ACO*), carnitine palmitoyltransferase 1 A (*CPT1A*), glucose-6-phosphatase (*G6P*) and phosphoenolpyruvate carboxykinase (*PEPCK*) increased (Fig. 7E–G; *P* < 0.05). These results suggest that CPE supplementation decreased lipid accumulation and increased insulin sensitivity and gluconeogenesis in the liver of dairy cows.

### Correlation analysis between rumen microbiome and rumen fermentation parameters, lactation performance, metabolic parameters and hepatic TG content

**Fig. 8:**
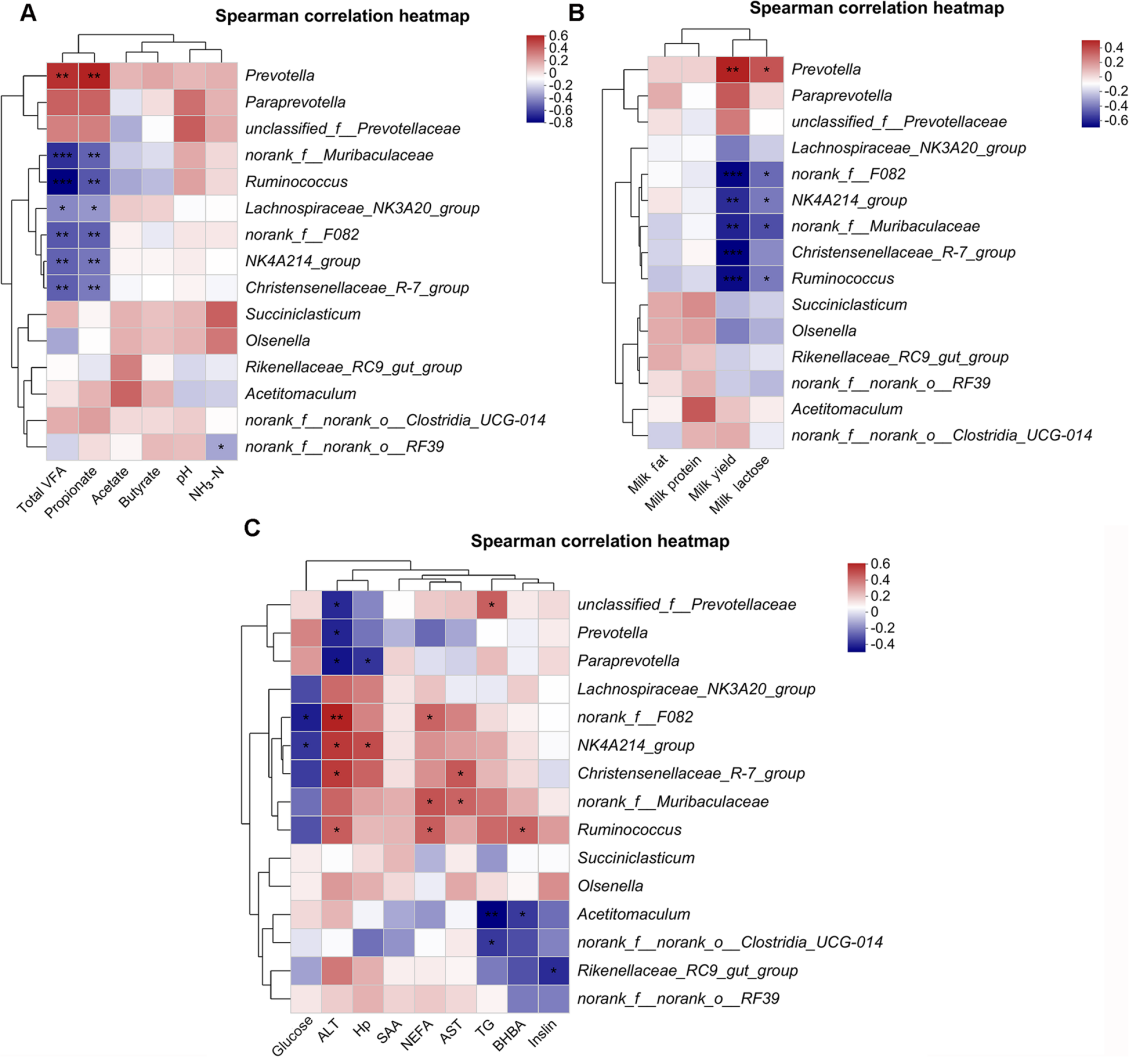
Correlation analysis between (**A**) different rumen microbiome and fermentation parameter, (**B**) different rumen microbiome and lactational performance and (**C**) different rumen microbiome and metabolic parameters. Red and blue indicate positive and negative correlations. *n* = 15 per group. ^*^P ˂ 0.05, ^**^P ˂ 0.01

Correlation analysis revealed that the concentration of total VFA and propionate were positively correlated with *Prevotella* (*P* < 0.01), and negatively correlated with *norank_f__Muribaculaceae* (*P* < 0.01), *Ruminococcus* (*P* < 0.01), *Lachnospiraceae_NK3A20_group* (*P* < 0.05), *norank_f__F082*, *NK4A214_group*, and *Christensenellaceae_R-7_group* (*P* < 0.01). Moreover, NH_3_-N concentration was found negatively correlated with *norank_f__norank_o__RF39* (Fig. 8A; *P* < 0.05). Milk yield and milk lactose content were positively correlated with *Prevotella* (*P* < 0.05), and negatively correlated with *norank_f__F082*, *NK4A214_group*, *norank_f__Muribaculaceae*, and *Ruminococcus* (*P* < 0.05). Milk yield was also negatively correlated with *Christensenellaceae_R-7_group* (Fig. 8B; *P* < 0.01).

Furthermore, glucose concentration was negatively correlated with *norank_f__F082* and *NK4A214_group* (*P* < 0.05). Additionally, NEFA levels were positively correlated with *norank_f__F082*, *norank_f__Muribaculaceae* and *Ruminococcus* (*P* < 0.05), while BHBA levels were positively correlated with *Ruminococcus* but negatively correlated with *Acetitomaculum* (*P* < 0.05). Insulin concentration was negatively correlated with *Rikenellaceae_RC9_gut_group* (*P* < 0.05). ALT levels were negatively correlated with *unclassified_f__Prevotellaceae*, *Prevotella* and *Paraprevotella* (*P* < 0.05), but positively correlated with *norank_f__F082* (*P* < 0.01), *NK4A214_group*, *Christensenellaceae_R-7_group* and *Ruminococcus* (*P* < 0.05). AST levels were positively correlated with *Christensenellaceae_R-7_group* and *norank_f__Muribaculaceae* (*P* < 0.05). Moreover, Hp concentration was negatively correlated with *Paraprevotella* (*P* < 0.05) and positively correlated with *NK4A214_group* (*P* < 0.05). Finally, hepatic TG content was positively correlated with *unclassified_f__Prevotellaceae* (*P* < 0.05) and negatively correlated with *Acetitomaculum* and *norank_f__norank_o__Clostridia_UCG-014* (Fig. 8C; *P* < 0.05).

## Discussion

Excessive NEB in dairy cows is associated with an increased risk of metabolic diseases such as ketosis and fatty liver, and is often accompanied by decreased milk production [35]. Rumen microorganisms play a crucial role in this context by converting complex plant-cell carbohydrates into VFA and microbial protein which are utilizable by cows to meet their energy needs [36]. Recent studies have shown that dietary supplementation with CPE increases milk yield and alters the diversity of the hindgut microbiota in dairy cows [30, 32]. In the present study, we observed that CPE supplementation led to alterations in rumen microbiota composition, increased ruminal VFA contents and enhanced milk yield of dairy cows. Moreover, CPE supplementation reduced adipose tissue lipolysis and hepatic lipid accumulation, while enhancing hepatic gluconeogenesis, which were associated with altered rumen microbiota, thus reducing the severity of adipose tissue inflammation during post-calving. These results suggest that CPE is a promising forage additive for improving rumen fermentation, energetic metabolism and lactation performance in dairy cows.

Despite Manach et al. [37] reported that CPE has a low bioavailability, Li et al. [29] observed that hesperidin, the main component of CPE, alleviated liver injury by regulating the intestinal microbiota. In our study, dietary supplementation with CPE altered the composition and diversity of the rumen microbiota of dairy cows in peripartum. In particular, the abundance of *Prevotella*, *Methanobrevibacter*, *norank_f__Bacteroidales_RF16_group*, and *Butyrivibrio* was increased in the rumen of CPE-fed dairy cows. Notably, Bacteroidales are the majority of VFA-producing rumen bacteria, while *Butyrivibrio* is known to degrade polysaccharides and ferment monosaccharides into VFA, predominantly propionic acid [38]. Consistent with these findings, CPE supplementation increased the ruminal contents of propionate, butyrate, acetate, and total VFA. In ruminants, approximately 90% of glucose is derived from gluconeogenesis, with 50%–60% originating from propionate [39]. Thus, the observed increase in serum glucose levels and decrease of serum NEFA and BHBA levels in CPE-fed cows post-parturition suggests that CPE supplementation elevated VFA production due to alterations in rumen microbiota composition contributes to alleviate NEB of dairy cows.

During the transition period, lipid mobilization from adipose tissue is an important compensatory mechanism that results in a significant release of NEFA into the circulation. Lipolysis is primarily driven by the action of ATGL and HSL, which convert TG into NEFA and glycerol [40, 41]. While NEFA serves as an essential energy substrate during NEB, elevated NEFA levels can disrupt liver function if not appropriately utilized [42]. In the present study, we observed reduced NEFA concentrations and lower protein expression of p-HSL and ATGL in dairy cows fed with CPE. Moreover, CPE supplementation decreased the expression of lipid droplet-associated proteins PLIN1 and CIDEC, which acts as barriers to the lipolysis by preventing ATGL and HSL access to lipid droplets [43]. It has been reported that the overexpression of CIDEC attenuated lipolysis in bovine and mouse adipocytes [44, 45]. Given that dairy cows experiencing severe energy deficits exhibit more extreme lipolysis than healthy cows [46], our findings indicate that CPE supplementation can alleviate NEB and reduce adipose tissue lipolysis in dairy cows.

Excessive lipolysis triggers inflammation and insulin resistance in adipocytes [47]. Dairy cows with ketosis exhibit sustained lipolysis, local inflammation, and insulin resistance of adipose tissue [48]. During the transition period, CPE supplementation alleviated adipose tissue lipolysis, inhibited the NF-κB signaling pathway, and increased insulin sensitivity. Previous study has reported that overexpression of PLIN1 increases ATGL expression and aggravates lipopolysaccharide-induced inflammatory responses in bovine adipocytes [49]. Additionally, inflammatory mediators such as TNF-α and IL-6 can induce lipolysis and reduce insulin sensitivity in adipocytes [50, 51]. This leads to a vicious circle where lipolysis activates the NF-κB signaling pathway, which induces proinflammatory cytokines overproduction and further exacerbates, lipolysis and insulin resistance. Thus, our findings suggest that CPE supplementation disrupts this vicious circle, thereby alleviating lipolysis, inflammatory response, and insulin resistance in adipocytes during early lactation in dairy cows.

In the present study, CPE was found to decrease lipid accumulation and increase insulin sensitivity in the liver of dairy cows. Hesperidin, the primary component of CPE, has been shown to alleviate hepatic steatosis and insulin resistance in human and rodents [29, 52, 53]. Previous study has reported that hesperidin alters the composition of the intestinal microbiota, downregulates genes involved in lipid synthesis, and upregulates genes involved in β-oxidation in the liver of mice fed a high-fat diet [29]. During early lactation, the uptake of NEFA in excess of metabolic requirements leads to NEFA re-esterification in the hepatocytes, resulting in hepatic steatosis in dairy cows. Accordingly, our findings indicate that CPE enhances VFA production by altering rumen microbiota composition and further alleviates NEB-induced lipid mobilization and hepatic steatosis.

During early lactation, the increased demand for glucose is met through enhanced hepatic gluconeogenesis. In dairy cows, propionate is the primary precursor for glucose synthesis, contributing more than 50% of the carbon required for gluconeogenesis [39]. Several studies have reported that propionate induces gluconeogenesis by upregulating the expression of genes involved in gluconeogenesis in the liver of dairy cows [54, 55]. Although serum VFA levels were not determined in the present study, our findings of increased ruminal VFA and upregulation of hepatic gluconeogenic gene expression suggest that CPE may have enhanced hepatic gluconeogenesis. Additionally, acetate, butyrate, and propionate have been reported to alleviate hepatic steatosis and insulin resistance in mice fed with a high-fat diet [56]. In palmitic acid-treated bovine hepatocytes, propionate improved endoplasmic reticulum stress and restored cell viability [13]. Thus, it is likely that the observed reduction of ALT and AST might be due to an enhanced VFA production in cows fed with CPE.

## Conclusion

In present study, it was found that dietary supplementation with CPE increased lactation performance, mitigated NEB, alleviated lipid mobilization and inflammation in adipose tissue of dairy cows during post-partum. These beneficial effects of CPE may be attributed to alterations in rumen microbiota composition and increased ruminal VFA contents. Thus, supplementing CPE in the diet of dairy cows can be a potential strategy to increase milk yield and improve energy metabolism during early lactation.

## Supplementary Information

Additional file 1: Table S1. Primers used during quantitative reverse-transcription PCR assay.

## Data Availability

The datasets produced or analyzed during the current study are available from the corresponding author on reasonable request.

## Abbreviations

ACC1: Acetyl CoA carboxylase 1
ACO: Acyl-CoA
AKT: Protein kinase B
ALT: Alanine aminotransferase
AST: Aspartate aminotransferase
ATGL: Adipose triglyceride lipase
BHBA: β-Hydroxybutyric acid
CASPASE-1: Cysteinyl aspartate specific proteinase
CIDEC: Cell death-inducing DNA fragmentation factor-α-like effector
CPE: Citrus peel extract
CPT1A: Carnitine palmitoyltransferase 1A
FAS: Fatty acid synthase
G6P: Glucose-6-phosphatase
Hp: Haptoglobin
HSL: Hormone-sensitive lipase
H&E: Hematoxylin–eosin
IκBα: Inhibitor of NF-κB
IL-1B: Interleukin 1B
IL-18: Interleukin 18
LDA: Linear discriminant analysis
LEfSe: LDA effect size
NEB: Negative energy balance
NEFA: Non-esterified fatty acid
NF-κB: Nuclear factor kappa-B
NH_3_-N: Ammonia nitrogen
NLRP3: NLR family pyrin domain containing protein 3
p-AKT: Phosphorylated-AKT
PCA: Principal component analysis
PCoA: Principal coordinate analysis
PEPCK: Phosphoenolpyruvate carboxykinase
PLIN1: Perilipin 1
PPARα: Peroxisome proliferator-activated receptor-α
SAA: Serum amyloid A protein
SOCS3: Suppressor of cytokine signaling 3
SREBP-1c: Sterol regulatory element-binding protein 1c
TG: Triglycerides
TNFA: Tumor necrosis factor-α
VFA: Volatile fatty acids

## References

[1] Caixeta LS, Omontese BO. Monitoring and improving the metabolic health of dairy cows during the transition period. Animals (Basel). 2021;11(2):352. 10.3390/ani11020352 PMC7911117 33572498

[2] Drackley JK. Biology of dairy cows during the transition period: the final frontier? J Dairy Sci. 1999;82:2259–73. 10575597 10.3168/jds.s0022-0302(99)75474-3

[3] Bertoni G, Trevisi E, Lombardelli R. Some new aspects of nutrition, health conditions and fertility of intensively reared dairy cows. Ital J Anim Sci. 2016;8:491–518.

[4] Herdt TH. Ruminant adaptation to negative energy balance. Influences on the etiology of ketosis and fatty liver. Vet Clin North Am Food Anim Pract. 2000;16:215–30. 11022337 10.1016/s0749-0720(15)30102-x

[5] Bobe G, Young JW, Beitz DC. Invited review: pathology, etiology, prevention, and treatment of fatty liver in dairy cows. J Dairy Sci. 2004;87:3105–24. 15377589 10.3168/jds.S0022-0302(04)73446-3

[6] Trevisi E, Minuti A. Assessment of the innate immune response in the periparturient cow. Res Vet Sci. 2018;116:47–54. 29223307 10.1016/j.rvsc.2017.12.001

[7] Kushibiki S, Hodate K, Shingu H, Obara Y, Touno E, Shinoda M, et al. Metabolic and lactational responses during recombinant bovine tumor necrosis factor-alpha treatment in lactating cows. J Dairy Sci. 2003;86:819–27. 12703618 10.3168/jds.S0022-0302(03)73664-9

[8] Bradford BJ, Mamedova LK, Minton JE, Drouillard JS, Johnson BJ. Daily injection of tumor necrosis factor-α increases hepatic triglycerides and alters transcript abundance of metabolic genes in lactating dairy cattle. J Nutr. 2009;139:1451–6. 19549751 10.3945/jn.109.108233

[9] Bergman EN. Energy contributions of volatile fatty acids from the gastrointestinal tract in various species. Physiol Rev. 1990;70:567–90. 2181501 10.1152/physrev.1990.70.2.567

[10] Gebreyesus G, Difford GF, Buitenhuis B, Lassen J, Noel SJ, Højberg O, et al. Predictive ability of host genetics and rumen microbiome for subclinical ketosis. J Dairy Sci. 2020;103:4557–69. 32197852 10.3168/jds.2019-17824

[11] Markantonatos X, Varga GA. Effects of monensin on glucose metabolism in transition dairy cows. J Dairy Sci. 2017;100:9020–35. 28888610 10.3168/jds.2016-12007

[12] Faniyi TO, Adegbeye MJ, Elghandour M, Pilego AB, Salem AZM, Olaniyi TA, et al. Role of diverse fermentative factors towards microbial community shift in ruminants. J Appl Microbiol. 2019;127:2–11. 30694580 10.1111/jam.14212

[13] Gao W, Fang Z, Lei L, Ju L, Jin B, Loor JJ, et al. Propionate alleviates palmitic acid-induced endoplasmic reticulum stress by enhancing autophagy in calf hepatic cells. J Dairy Sci. 2021;10:9316–26. 10.3168/jds.2020-19969 34001357

[14] May KS, Hartigh LJ. Modulation of adipocyte metabolism by microbial short-chain fatty acids. Nutrients. 2021;13:3666. 34684670 10.3390/nu13103666 PMC8538331

[15] Minuti A, Palladino A, Khan MJ, Alqarni S, Agrawal A, Piccioli-Capelli F, et al. Abundance of ruminal bacteria, epithelial gene expression, and systemic biomarkers of metabolism and inflammation are altered during the peripartal period in dairy cows. J Dairy Sci. 2015;98:8940–51. 26409956 10.3168/jds.2015-9722

[16] Xiang K, Li S, Tuniyazi M, Mu R, Wang Y, Zhang N, et al. Changes in the rumen microbiota community in ketosis cows during propylene glycol treatment. Food Funct. 2022;13:7144–56. 35699056 10.1039/d1fo03800a

[17] Schären M, Frahm J, Kersten S, Meyer U, Hummel J, Breves G, et al. Interrelations between the rumen microbiota and production, behavioral, rumen fermentation, metabolic, and immunological attributes of dairy cows. J Dairy Sci. 2018;101:4615–37. 29454699 10.3168/jds.2017-13736

[18] Wang X, Li X, Zhao C, Hu P, Chen H, Liu Z, et al. Correlation between composition of the bacterial community and concentration of volatile fatty acids in the rumen during the transition period and ketosis in dairy cows. Appl Environ Microbiol. 2012;78:2386–92. 22267666 10.1128/AEM.07545-11 PMC3302620

[19] Wang Q, Cui Y, Indugu N, Loor JJ, Jiang Q, Yu Z, et al. Integrated meta-omics analyses reveal a role of ruminal microorganisms in ketone body accumulation and ketosis in lactating dairy cows. J Dairy Sci. 2023;106:4906–17. 37296048 10.3168/jds.2022-22282

[20] Kholif AE, Hassan AA, El Ashry GM, Bakr MH, El-Zaiat HM, Olafadehan OA, et al. Phytogenic feed additives mixture enhances the lactational performance, feed utilization and ruminal fermentation of friesian cows. Anim Biotechnol. 2021;32:708–18. 32248772 10.1080/10495398.2020.1746322

[21] Rao ZX, Tokach MD, Woodworth JC, DeRouchey JM, Goodband RD, Gebhardt JT. Effects of various feed additives on finishing pig growth performance and carcass characteristics: a review. Animals (Basel). 2023;13:200. 10.3390/ani13020200 PMC9854424 36670740

[22] Lopreiato V, Mezzetti M, Cattaneo L, Ferronato G, Minuti A, Trevisi E. Role of nutraceuticals during the transition period of dairy cows: a review. J Anim Sci Biotechnol. 2020;11:96. 32864127 10.1186/s40104-020-00501-x PMC7450574

[23] Gessner DK, Koch C, Romberg FJ, Winkler A, Dusel G, Herzog E, et al. The effect of grape seed and grape marc meal extract on milk performance and the expression of genes of endoplasmic reticulum stress and inflammation in the liver of dairy cows in early lactation. J Dairy Sci. 2015;98:8856–68. 26409958 10.3168/jds.2015-9478

[24] Wang Y, Nan X, Zhao Y, Jiang L, Wang H, Hua D, et al. Dietary supplementation with inulin improves lactation performance and serum lipids by regulating the rumen microbiome and metabolome in dairy cows. Anim Nutr. 2021;7:1189–204. 34754961 10.1016/j.aninu.2021.09.007 PMC8556608

[25] Mas-Capdevila A, Teichenne J, Domenech-Coca C, Caimari A, Del Bas JM, Escote X, et al. Effect of hesperidin on cardiovascular disease risk factors: the role of intestinal microbiota on hesperidin bioavailability. Nutrients. 2020;12:1488. 32443766 10.3390/nu12051488 PMC7284956

[26] Li R, Cai L, Xie XF, Yang F, Li J. Hesperidin suppresses adjuvant arthritis in rats by inhibiting synoviocyte activity. Phytother Res. 2010;24(Suppl 1):S71–6. 19585485 10.1002/ptr.2906

[27] Choi SS, Lee SH, Lee KA. A comparative study of hesperetin, hesperidin and hesperidin glucoside: antioxidant, anti-inflammatory, and antibacterial activities in vitro. Antioxidants (Basel). 2022;11(8):1618. 10.3390/antiox11081618 PMC9405481 36009336

[28] Matsuo Y, Akita K, Taguchi H, Fujii S, Yoshie-Stark Y, Araki T. Utilization and evaluation of *Citrus natsudaidai* peel waste as a source of natural food additives. Food Chem. 2022;373:131464. 34741966 10.1016/j.foodchem.2021.131464

[29] Li X, Yao Y, Wang Y, Hua L, Wu M, Chen F, et al. Effect of hesperidin supplementation on liver metabolomics and gut microbiota in a high-fat diet-induced NAFLD mice model. J Agric Food Chem. 2022;70:11224–35. 36048007 10.1021/acs.jafc.2c02334

[30] Zhao Y, Yu S, Zhao H, Li L, Li Y, Tu Y, et al. Lipidomic profiling using GC and LC-MS/MS revealed the improved milk quality and lipid composition in dairy cows supplemented with citrus peel extract. Food Res Int. 2022;161:111767. 36192874 10.1016/j.foodres.2022.111767

[31] Yu S, Zhao Y, Li L, Zhao H, Liu M, Jiang L. Flavonoids from citrus peel display potential synergistic effects on inhibiting rumen methanogenesis and ammoniagenesis: a microbiome perspective. Environ Sci Pollut Res Int. 2024;31:21208–23. 38383931 10.1007/s11356-024-32509-5

[32] Zhao Y, Yu S, Zhao H, Li L, Li Y, Liu M, et al. Integrated multi-omics analysis reveals the positive leverage of citrus flavonoids on hindgut microbiota and host homeostasis by modulating sphingolipid metabolism in mid-lactation dairy cows consuming a high-starch diet. Microbiome. 2023;11:236. 37880759 10.1186/s40168-023-01661-4 PMC10598921

[33] Broderick GA, Kang JH. Automated simultaneous determination of ammonia and total amino acids in ruminal fluid and in vitro media. J Dairy Sci. 1980;63:64–75. 7372898 10.3168/jds.S0022-0302(80)82888-8

[34] Liu C, Zhao D, Ma W, Guo Y, Wang A, Wang Q, et al. Denitrifying sulfide removal process on high-salinity wastewaters in the presence of *Halomonas* sp. Appl Microbiol Biotechnol. 2016;100:1421–6. 26454867 10.1007/s00253-015-7039-6

[35] Martens H. Invited review: increasing milk yield and negative energy balance: a gordian knot for dairy cows? Animals (Basel). 2023;13:3097. 10.3390/ani13193097 PMC10571806 37835703

[36] Xue MY, Sun HZ, Wu XH, Liu JX, Guan LL. Multi-omics reveals that the rumen microbiome and its metabolome together with the host metabolome contribute to individualized dairy cow performance. Microbiome. 2020;8:64. 32398126 10.1186/s40168-020-00819-8 PMC7218573

[37] Manach C, Morand C, Gil-Izquierdo A, Bouteloup-Demange C, Rémésy C. Bioavailability in humans of the flavanones hesperidin and narirutin after the ingestion of two doses of orange juice. Eur J Clin Nutr. 2003;57:235–42. 12571654 10.1038/sj.ejcn.1601547

[38] Palevich N, Kelly WJ, Leahy SC, Denman S, Altermann E, Rakonjac J, et al. Comparative genomics of rumen butyrivibrio spp. uncovers a continuum of polysaccharide-degrading capabilities. Appl Environ Microbiol. 2019;86:e01993–19. 31653790 10.1128/AEM.01993-19 PMC6912079

[39] Reynolds CK, Huntington GB, Tyrrell HF, Reynolds PJ. Net metabolism of volatile fatty acids, D-beta-hydroxybutyrate, nonesterifield fatty acids, and blood gasses by portal-drained viscera and liver of lactating Holstein cows. J Dairy Sci. 1988;71:2395–405. 3141488 10.3168/jds.s0022-0302(88)79824-0

[40] Park JG, Xu X, Cho S, Hur KY, Lee MS, Kersten S, et al. CREBH-FGF21 axis improves hepatic steatosis by suppressing adipose tissue lipolysis. Sci Rep. 2016;6:27938. 27301791 10.1038/srep27938 PMC4908383

[41] Dong J, Yue K, Loor JJ, Aboragah A, Li G, Chen L, et al. Increased adipose tissue lipolysis in dairy cows with fatty liver is associated with enhanced autophagy activity. J Dairy Sci. 2022;105:1731–42. 34998548 10.3168/jds.2021-20445

[42] Li Y, Ding HY, Wang XC, Feng SB, Li XB, Wang Z, et al. An association between the level of oxidative stress and the concentrations of NEFA and BHBA in the plasma of ketotic dairy cows. J Anim Physiol Anim Nutr (Berl). 2016;100:844–51. 27079290 10.1111/jpn.12454

[43] Sohn JH, Lee YK, Han JS, Jeon YG, Kim JI, Choe SS, et al. Perilipin 1 (Plin1) deficiency promotes inflammatory responses in lean adipose tissue through lipid dysregulation. J Biol Chem. 2018;293:13974–88. 30042231 10.1074/jbc.RA118.003541 PMC6130955

[44] Fan M, Du X, Chen X, Bai H, Loor JJ, Shen T, et al. Inhibition of cell death inducing DNA fragmentation factor-α-like effector c (CIDEC) by tumor necrosis factor-α induces lipolysis and inflammation in calf adipocytes. J Dairy Sci. 2021;104:6134–45. 33685683 10.3168/jds.2020-19319

[45] Shamsi BH, Ma C, Naqvi S, Xiao Y. Effects of pioglitazone mediated activation of PPAR-γ on CIDEC and obesity related changes in mice. PLoS ONE. 2014;9:e106992. 25210844 10.1371/journal.pone.0106992 PMC4161383

[46] McFadden JW. Review: lipid biology in the periparturient dairy cow: contemporary perspectives. Animal. 2020;14:s165–75. 32024571 10.1017/S1751731119003185

[47] Contreras GA, Strieder-Barboza C, De Koster J. Symposium review: modulating adipose tissue lipolysis and remodeling to improve immune function during the transition period and early lactation of dairy cows. J Dairy Sci. 2018;101:2737–52. 10.3168/jds.2017-13340 29102145

[48] Sordillo LM, Raphael W. Significance of metabolic stress, lipid mobilization, and inflammation on transition cow disorders. Vet Clin North Am Food Anim Pract. 2013;29:267–78. 23809891 10.1016/j.cvfa.2013.03.002

[49] Zhang S, Liu G, Xu C, Liu L, Zhang Q, Xu Q, et al. Perilipin 1 mediates lipid metabolism homeostasis and inhibits inflammatory cytokine synthesis in bovine adipocytes. Front Immunol. 2018;9:467. 29593725 10.3389/fimmu.2018.00467 PMC5854662

[50] Du X, Liu M, Tai W, Yu H, Hao X, Loor JJ, et al. Tumor necrosis factor-α promotes lipolysis and reduces insulin sensitivity by activating nuclear factor kappa B and c-Jun N-terminal kinase in primary bovine adipocytes. J Dairy Sci. 2022;105:8426–38. 35965124 10.3168/jds.2022-22009

[51] van Hall G, Steensberg A, Sacchetti M, Fischer C, Keller C, Schjerling P, et al. Interleukin-6 stimulates lipolysis and fat oxidation in humans. J Clin Endocrinol Metab. 2003;88:3005–10. 12843134 10.1210/jc.2002-021687

[52] Cheraghpour M, Imani H, Ommi S, Alavian SM, Karimi-Shahrbabak E, Hedayati M, et al. Hesperidin improves hepatic steatosis, hepatic enzymes, and metabolic and inflammatory parameters in patients with nonalcoholic fatty liver disease: a randomized, placebo-controlled, double-blind clinical trial. Phytother Res. 2019;33:2118–25. 31264313 10.1002/ptr.6406

[53] Atta IS, Elnady MR, Alghamdi AG, Alghamdi AH, Aboulata AA, Shatla IM. Assessing the hepatoprotective effects of hesperidin on liver-associated disorders in albino rats with experimentally induced obesity and type II diabetes: a histological and biochemical study. Heliyon. 2023;9:e16031. 37215885 10.1016/j.heliyon.2023.e16031 PMC10196525

[54] Caputo Oliveira R, Erb SJ, Pralle RS, Holdorf HT, Seely CR, White HM. Postpartum supplementation with fermented ammoniated condensed whey altered nutrient partitioning to support hepatic metabolism. J Dairy Sci. 2020;103:7055–67. 32534927 10.3168/jds.2019-17790

[55] Zhang Q, Koser SL, Bequette BJ, Donkin SS. Effect of propionate on mRNA expression of key genes for gluconeogenesis in liver of dairy cattle. J Dairy Sci. 2015;98:8698–709. 26409969 10.3168/jds.2015-9590

[56] Shimizu H, Masujima Y, Ushiroda C, Mizushima R, Taira S, Ohue-Kitano R, et al. Dietary short-chain fatty acid intake improves the hepatic metabolic condition via FFAR3. Sci Rep. 2019;9:16574. 31719611 10.1038/s41598-019-53242-x PMC6851370

