# Supplementary material for: Dietary supplementation with citrus peel extract in transition period improves rumen microbial composition and ameliorates energy metabolism and lactation performance of dairy cows
Source: J Anim Sci Biotechnol. 2024 Nov 9;15:152. doi: 10.1186/s40104-024-01110-8 (PMC11549748; doi:10.1186/s40104-024-01110-8)
Supplement: Supplementary file 1 — Additional file 1: Table S1. Primers used during quantitative reverse-transcription PCR assay. [file 40104_2024_1110_MOESM1_ESM.docx]

**Supplemental material**

**Table S1** Primers used during quantitative reverse-transcription PCR assay

| **Gene** | **Primers (5′→3′)** | **GenBank number** | **Length, bp** | **Annealing temperature, °C** |
| --- | --- | --- | --- | --- |
| *NLRP3* | \| For: CTTTCTGGACTCTGACCGGG \| \| --- \| \| Rev: ATGCCTTCTCTTCCCCGTTG \| | NM_001102219.1 | 229 | 60 |
| *SOCS3* | For: GCCACTCTCCAACATCTCTGT  Rev: TCCAGGAACTCCCGAATGG | NM_174466.2 | 98 | 60 |
| *CASPASE-1* | For: CAGTGGTCCCTCCTTTTCCAG  Rev: TCCACCTTGTATCCCAGACCT | XM_024975700.1 | 286 | 60 |
| *TNFA* | For: TCTACCAGGGAGGAGTCTTCCA  Rev: GTCCGGCAGGTTGATCTCA | NM_173966.3 | 68 | 60 |
| *IL-18* | For: CTATTGAGCACAGGCATAAAGATG  Rev: TGATCTGATTCCAGGTCTTCATCA | NM_001304989.2 | 119 | 60 |
| *IL-1B* | For: CCTCGGTTCCATGGGAGATG  Rev: AACTCGTCGGAGGACGTTTC | NM_001045889.2 | 94 | 60 |
| *SREBP-1c* | For: GACACCACCAGCATCAACCACG  Rev: CAGCCCATTCATCAGCCAGACC | NM_  001113302.1 | 117 | 60 |
| *ACC1* | For: TCCTGCTGCTATTGCTACTCCA  Rev: CAGTCCCCGCACTCACATAA | NM_  174224.2 | 95 | 60 |
| *FAS* | For: ACAGCCTCTTCCTGTTTGACG  Rev: CTCTGCACGATCAGCTCGAC | NM_  001012669.1 | 144 | 60 |
| *PPARA* | For: TCAGATGGCTCCGTTATT  Rev: CCCGCAGATCCTACACT | NM_001034036.1 | 132 | 60 |
| *ACO* | For: TAAGCCTTTGCCAGGTATT  Rev: ATGGTCCCGTAGGTCAG | NC_015500.1 | 189 | 60 |
| *CPT1A* | For: ACGCCGTGAAGTATAACCCT  Rev: CCAAAAATCGCTTGTCCCTT | NM_001304989.2 | 119 | 60 |
| *G6P* | For: AGCAAGTGGTTCCCGTTTC  Rev: ACCCAGGCGAGGCAGTA | NM_001076124.2 | 179 | 60 |
| *PEPCK* | For: AAGTACCTTGAGGAGCAAGTGAA  Rev: GGTGCGTTGTATGGATTGGA | NM_174737.2 | 133 | 60 |
| *GAPDH* | For: GGCGTGAACCACGAGAAGTATAA  Rev: CCTCCACGATGCCAAAGTG | NM_001034034.2 | 118 | 60 |

*NLRP3* NLR family pyrin domain containing protein 3, *SOCS3* Suppressor of cytokine signaling 3, *CASPASE-1* Cysteinyl aspartate specific proteinase, *TNFA* Tumor necrosis factor-α, *IL-18* Interleukin 18, *IL-1B* Interleukin 1B, *SREBP1c* Sterol regulatory element-binding protein-1c, *ACC1* Acetyl CoA carboxylase 1, *FAS* Fatty acid synthase, *PPARA* Peroxisome proliferator-activated receptor-α, *ACO* Acyl-CoA oxidase, *CPTIA* Carnitine palmitoyltransferase 1A, *G6P* Glucose-6-phosphatase, *PEPCK* Phosphoenolpyruvate carboxykinase, *GAPDH* Glyceraldehyde-3-phosphate dehydrogenase
